# ciRS-7 Promotes the Proliferation and Migration of Papillary Thyroid Cancer by Negatively Regulating the miR-7/Epidermal Growth Factor Receptor Axis

**DOI:** 10.1155/2020/9875636

**Published:** 2020-06-21

**Authors:** Jun-ya Han, Si Guo, Na Wei, Rui Xue, Wencai Li, Gang Dong, Jianhua Li, Xiangyu Tian, Chao Chen, Sen Qiu, Tong Wang, Qiankun Xiao, Chenguang Liu, Jingjing Xu, Kui-sheng Chen

**Affiliations:** ^1^Department of Pathology, The First Affiliated Hospital of Zhengzhou University, No. 1 Jian She Dong Avenue, Zhengzhou, China; ^2^Henan Key Laboratory of Tumor Pathology, No. 1 Jian She Dong Avenue, Zhengzhou, China; ^3^Academy of Medical Sciences, Zhengzhou University, No. 100 Ke Xue Avenue, Zhengzhou, China; ^4^Department of Medical Laboratory, Henan Provincial People's Hospital, Zhengzhou, Henan, China; ^5^Department of Medical Laboratory of Central China Fuwai Hospital, Central China Fuwai Hospital of Zhengzhou University, Zhengzhou, Henan, China; ^6^Medical Research Center, The First Affiliated Hospital of Zhengzhou University, No. 1 Jian She Dong Avenue, Zhengzhou, China; ^7^Department of Ultrasound, The First Affiliated Hospital of Zhengzhou University, No. 1 Jian She Dong Avenue, Zhengzhou, China; ^8^Thyroid Surgery Department of the First Affiliated Hospital of Zhengzhou University, No. 1 Jian She Dong Avenue, Zhengzhou, China

## Abstract

**Purpose:**

The incidence of papillary thyroid cancer (PTC) is increasing, and traditional diagnostic methods are unsatisfactory. Therefore, identifying novel prognostic markers is very important. ciRS-7 has been found to play an important role in many cancers, but its role in PTC has not been reported. This study was performed to evaluate the biological role and mechanism of ciRS-7 in PTC. *Material and Methods*. The expression of ciRS-7 in PTC tissues and the matched adjacent tissues was determined by quantitative reverse transcription polymerase chain reaction (qRT-PCR). The PTC cell lines (TPC-1 and BCPAP) were used to evaluate the role of ciRS-7. ciRS-7-siRNA and overexpression plasmid were constructed and transfected into PTC cells. A CCK-8 assay and colony formation assay were performed to explore the effects of ciRS-7 on cell proliferation. Annexin V/PI staining and FACS detection were used to detect cell apoptosis. Wound healing assay was performed to detect cell migration. A transwell assay was conducted to explore the effects of ciRS-7 on invasion and migration. Western blotting was performed to evaluate protein expression. The luciferase reporter system was used to determine the underlying mechanism of miR-7.

**Result:**

ciRS-7 was highly expressed in PTC tissues and cell lines compared with the corresponding controls. In vitro study showed that ciRS-7 silencing suppressed proliferation, migration, and invasion of TPC-1 and BCPAP. Mechanistically, the effects of ciRS-7 on invasion and migration may be related to epithelial-mesenchymal transition (EMT). ciRS-7 silencing could attenuate effects on PTC cells induced by miR-7 knockdown. Epidermal growth factor receptor (EGFR), which was demonstrated to be a target of miR-7, decreased significantly in ciRS-7-siRNA PTC cells. Overexpression of EGFR also attenuated effects of PTC cells induced by silencing ciRS-7.

**Conclusion:**

ciRS-7 was significantly upregulated in PTC tissues, and it promoted the progression of PTC by regulating the miR-7/EGFR axis. ciRS-7 is a promising prognostic biomarker and therapeutic target in PTC.

## 1. Introduction

Papillary thyroid cancer (PTC) is the most prevalent thyroid malignancy, accounting for 80–85% of all thyroid cancers [1]. The incidence of PTC has steadily and rapidly increased over the past 40 years. Particularly, in the past five years, the incidence has increased faster than that of any other type of cancer. Although PTC has a good prognosis, 30% of patients may develop persistent disease or relapse. Somatic BRAFV600E mutation is used for diagnosing PTC, but its prognostic effect remains controversial [2–4]. Most PTCs are surgically removed and treated with adjuvant radioactive iodine. Nevertheless, a fraction of PTCs progresses to metastatic disease and/or does not respond to adjuvant radioactive iodine therapy; the prognosis of these cases is poor, with a 10-year survival rate of 10% [5–7]. New prognostic markers useful for optimizing therapy and long-time follow-up care must be identified. Circular RNAs are a class of RNA molecules without 5′ and 3′ ends, cap structure, or poly-A tail structure. Because of their circular configuration, they are protected from degradation by RNases [8]. This is advantageous compared to the use of linear RNA as markers for diagnosis and prognosis [9]. Recent studies indicated that circRNAs play a critical regulatory function in multiple physiological and pathological processes. For example, circRNA_102958 sponges miR-585 to promote the tumorigenesis of colorectal cancer [10]. In nucleus pulposus tissues of intervertebral disc degeneration, circRNA_104670 sponges miR-17-3p to regulate matrix metalloproteinase-2 [11]. In particular, the role of circRNAs as miRNA “sponges” in tumorigenesis and development has been widely investigated [9]. Unfortunately, although emerging evidences indicated that circRNAs play a critical regulatory function in proliferation and invasion of cancer cells, the study about functions of circRNA in PTC has just begun. Moreover, whether circRNAs in PTC sponge to miRNAs and regulate the expression level of downstream genes is also unclear.

Few evidence about functions and mechanisms of circRNAs exists at present.

ciRS-7, also named as cerebellar degeneration-related protein 1 antisense RNA (CDR1as) or CDR1NAT, comprises 1500 nucleotides and is predominantly present in the brain of humans and mice [12]. It plays a significant role in the diagnosis, prognosis, and treatment of various malignant tumors, as it targets microRNA-7 (miR-7) in multiple tumor types [13]. In this study, we found that ciRS-7 expression was significantly elevated in PTC tissues and cell lines compared with their normal controls. Upregulation of ciRS-7 is closely associated with poor prognoses. Mechanically, we demonstrated that ciRS-7 could act as a sponge of miR-7 to upregulate the level of EGFR and trigger PTC proliferation, invasion, and migration. Our data revealed that ciRS-7 overexpression promotes PTC progression via the miR-7/EGFR axis. ciRS-7 may be a prognostic marker and therapeutic target for PTC.

## 2. Materials and Methods

### 2.1. Human Tissues

A total of 17 patients at the First Affiliated Hospital of Zhengzhou University (Zhengzhou, China) with PTC confirmed pathologically were enrolled in this study. Fresh thyroid carcinoma tissues and adjacent normal thyroid tissues located more than 3 cm away from the cancer site were collected and immediately frozen in liquid nitrogen. No patient had been administered therapy. The research protocol was approved by the Ethics Committees of the First Affiliated Hospital of Zhengzhou University. All patients provided informed written consent for all procedures. The clinicopathological parameters were archived from the medical record.

### 2.2. Cell Culture and Transfection

Human PTC cell TPC-1 and BCPAP and human thyroid follicular epithelial cell line Nthy-ori 3-1 were acquired from American Type Culture Collection (Manassas, VA, USA). They were cultured in Roswell Park Memorial Institute (RPMI) 1640 medium containing 10% fetal bovine serum (Gibco, Grand Island, NY, USA), 100 U/mL penicillin, and 100 *μ*L/mL streptomycin. They were incubated in a humidified chamber in an atmosphere of 5% CO_2_ at 37°C. ciRS-7-siRNA, ciRS-overexpressed plasmid, miR-7 mimic, miR-7 inhibitor, EGFR-overexpressed plasmid, and the empty vectors were obtained from Genema (Shanghai, China). Cell transfections were performed using Lipofectamine 2000 reagent (Invitrogen) according to the manufacturer's instructions.

### 2.3. RNA Extraction and Quantitative qRT-PCR

The total RNA from tissues or cells was isolated using TRIzol reagent (Beyotime, Shanghai, China) according to the manufacturer's instructions. Complementary DNA (cDNA) was generated by reverse transcription using a PrimeScript™ RT reagent kit (Takara, Shiga, Japan). Quantitative PCR was performed with TB Green Premix Ex Taq™ II (TaKaRa) on an ABI Prism 7900 sequencer (Applied Biosystems, Foster City, CA, USA). U6 was used to normalize the level of microRNA-7 expression, while GAPDH was used as an internal control for the determination ciRS-7. The data were analyzed using the 2^-*ΔΔ*CT^ method. Primer sequences were synthesized by SanYa (Shanghai, China) as follows: ciRS-7, R (reverse): 5′-TGTATCCAGAGTTACTTCCAGTGT-3′, F (forward): 5′-TCAGCAGTTTCATCTTCTTCTTCA-3′; microRNA-7, RT (reverse transcription): 5′-GTCGTATCCAGTGCAGGGTCCGAGGTATTCGCACTGGAT CGACAACAAC-3′, R (reverse): 5′-CGCGCGTGGAAGACTAGTGATTTT-3′, F (forward): 5′-AGTGCAGGGTCCGAGGTATT-3′.

### 2.4. Western Blot Assay

All the cells and tissues were lysed in RIPA buffer containing protease and phosphatase inhibitors. Proteins were separated by 10% SDS-polyacrylamide gels and transferred to polyvinylidene difluoride membrane (Bio-Rad, Hercules, CA, USA). Membranes were incubated at 4°C with anti-EGFR (1 : 1000; Abcam, Cambridge, UK), anti-E-cadherin (1 : 1000; Abcam), anti-vimentin (1 : 1000; Abcam), and anti-GAPDH (1 : 5000; Abcam) antibodies overnight. Subsequently, membranes were incubated with HRP-conjugated secondary antibodies. Protein expression levels were visualized using electrochemiluminescence substrates (Pierce, Rockford, IL, USA).

### 2.5. Cell Proliferation Assay and Colony Formation Assay

Cell proliferation was determined using a CCK-8 assay kit (Dojindo, Kumamoto, Japan) according to the manufacturer's instructions. Cells were seeded (100 *μ*L; 2 × 10^3^ cells per well) into 96-well plates, and CCK-8 was added at 0, 24, 48, and 72 h. Absorbance was measured at 450 nM using an enzyme-labeling instrument (Thermo Fisher Scientific) afterward. For colony formation assay, cells were seeded (500 cells per well) into six-well plates and incubated for 10 days. The colonies were stained and observed under a microscope.

### 2.6. Cell Apoptosis Assay

After transfection for 48 h, cells were collected for cell apoptosis detection. The apoptosis was measured using the PE Annexin V Apoptosis Detection Kit (BD Biosciences) according to the manufacturer's instructions. FACSCalibur was employed to detect the cell apoptosis. FACSDiva was applied to analyze the data.

### 2.7. Scratch Test

Cells were seeded into six-well plates to detect the wound healing capabilities. When the cells covered 80–90% of the dish, a 100 *μ*L pipette tip was used to make four scratches at the same width in each well. PBS was used to wash away the cells removed during scratching. Next, the cells were cultured in fresh culture medium in an incubator. An inverted microscope was used to observe the migration distance of cells into the scratch area at 0, 6, 24, and 36 h. The assay was repeated three times.

### 2.8. Transwell Migration and Invasion

Transwell migration assays were performed using a transwell chamber (Corning, China). Transfected cells in serum-free medium were seeded in the upper transwell chamber, and 500 *μ*L RPMI solution that included 20% fetal bovine (FBS) serum was added into the lower transwell chamber. After being cultured for 24 hours, cells were fixed with paraformaldehyde and stained using crystal violet. An inverted light microscope was used to quantify cell migration, and views were randomly observed to obtain the average results. Invasion assay was conducted in accordance with the above procedures except that the bottom membranes were coated with the diluted Matrigel.

### 2.9. Dual-Luciferase Reporter Assay

A dual-luciferase reporter assay was performed in stable PTC cells. Wild-type (WT-EGFR) and mutant (MUT-EGFR) plasmids were constructed by Genema (Shanghai, China). Four groups including microRNA-7/EGFR-WT-3′UTR, NC/EGFR-WT-3′UTR, microRNA-7/EGFR-MUT-3′UTR, and NC/EGFR-MUT-3′UTR were established. After transfection for 48 h, cells were collected for dual-luciferase activity assay using a Promega Dual-Luciferase Reporter Assay System (Promega, Madison, WI, USA).

### 2.10. Statistical Analysis

All statistical analyses were performed using SPSS 20.0 software (SPSS, Inc., Chicago, IL, USA). Significant differences between groups were estimated using a two-tailed Student's *t*-test, or the Wilcoxon test, as appropriate. Variables with a *P* < 0.05 in univariate analyses were subsequently used for multivariate analyses based on Cox regression analyses. Two-tailed *P* values were calculated, and statistical significance was set at *P* < 0.05.

## 3. Results

### 3.1. The Expression of ciRS-7 in PTC Tissues and Cell Lines

**Figure 1:**
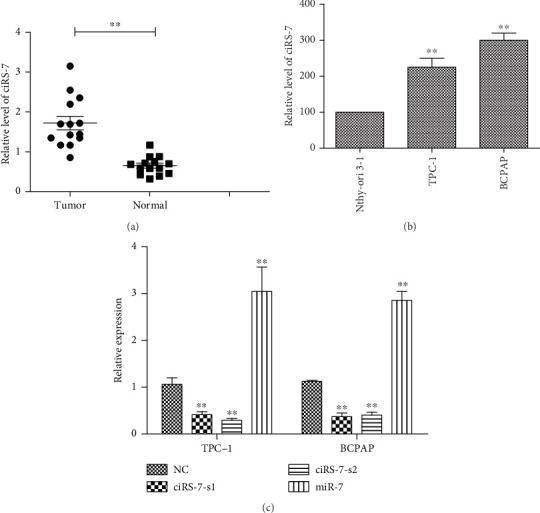
The expression of ciRS-7 in PTC. The expression of ciRS-7 in 17 pairs of matched PTC tissues and adjacent normal tissues was measured by qRT-PCR (a). The expression of ciRS-7 in PTC cell (TPC-1 and BCPAP) and human thyroid epithelial cell line Nthy-ori3-1 was measured by qRT-PCR (b). The expression of ciRS-7 was knocked down by transfection of ciRS-7-siRNA and verification by qRT-PCR. The expression of miR-7 was upregulated by ciRS-7 silencing (c). ^∗^*P* < 0.05, ^∗∗^*P* < 0.01, and ^∗∗∗^*P* < 0.001.

To analyze the role of ciRS-7 in PTC, we measured the expression of ciRS-7 in 17 pairs of PTC and their normal counterparts. ciRS-7 was highly expressed in PTC tissues (Figure 1(a)). ciRS-7 levels were also consistently higher in PTC cell lines TPC-1 and BCPAP than human thyroid epithelial cell line Nthy-ori 3-1 (Figure 1(b)). In addition, while the overexpression of ciRS-7 was significantly correlated with large tumor size (*P* = 0.015) and lymph metastasis (*P* = 0.022), it was not correlated with either age (*P* ≥ 0.05) or gender (*P* ≥ 0.05) (Table 1).

### 3.2. Promotive Effects of ciRS-7 in PTC

**Figure 2:**
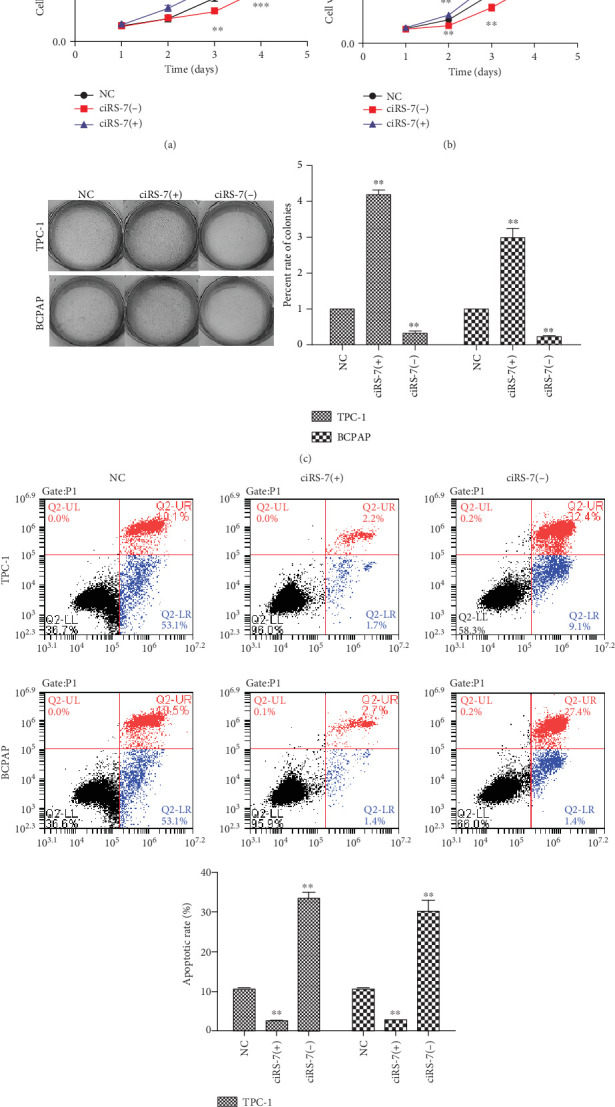
The effects of ciRS-7 on PTC cell proliferation and apoptosis. Cell proliferation of PTC cells (TPC-1 and BCPAP) after ciRS-7-siRNA, ciRS-7 overexpression, or negative control siRNA transfection was evaluated by CCK-8 assay (a, b). Images of colony formation assay using TPC-1 and BCPAP cells and quantification analysis of colony numbers (c). Annexin V/PI staining and FACS detection were used to detect cell apoptosis (d). ^∗^*P* < 0.05, ^∗∗^*P* < 0.01, and ^∗∗∗^*P* < 0.001.

**Figure 3:**
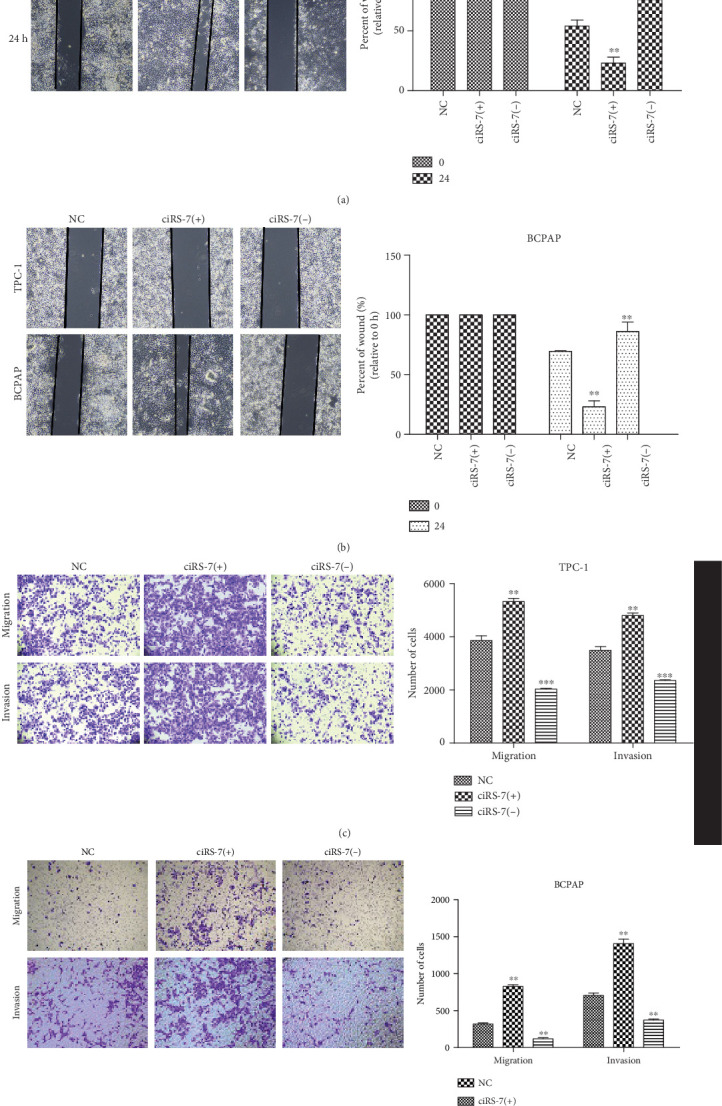
The effects of ciRS-7 on PTC cell migration and invasion. Wound healing assay was conducted to assess the cell migration after ciRS-7-siRNA, ciRS-7 overexpression, or negative control siRNA transfection (a, b). Transwell assays were conducted to assess the cell migration and invasion abilities of PTC cells (TPC-1 and BCPAP) after transfection (c, d). ^∗^*P* < 0.05, ^∗∗^*P* < 0.01, and ^∗∗∗^*P* < 0.001.

To explore the functions of ciRS-7, two short interfering (ciRS-7-s1 and ciRS-7-s2) (Supplementary Materials (available here)) vectors were transfected into TPC-1 and BCPAP cell lines (Figure 1(c)). CCK-8 assay showed that ciRS-7 downexpression significantly inhibits the in vitro proliferation of TPC-1 and BCPAP (Figures 2(a) and 2(b)). This was confirmed by colony formation assay (Figure 2(c)) and flow cytometry (Figure 2(d)). In addition, decreased invasion and migration in vitro were observed in ciRS-7-downexpressed TPC-1 and BCPAP cells using wound healing assay (Figures 3(a) and 3(b)) and transwell assays (Figures 3(c) and 3(d)). Oppositely, overexpression of ciRS-7 promoted the proliferation, migration, and invasion in PTC cell lines (Figures 2 and 3). Collectively, the above results demonstrated that ciRS-7 plays an oncogene role in PTC.

### 3.3. ciRS-7 Facilitated Epithelial-Mesenchymal Transition (EMT) of PTC Cells

**Figure 4:**
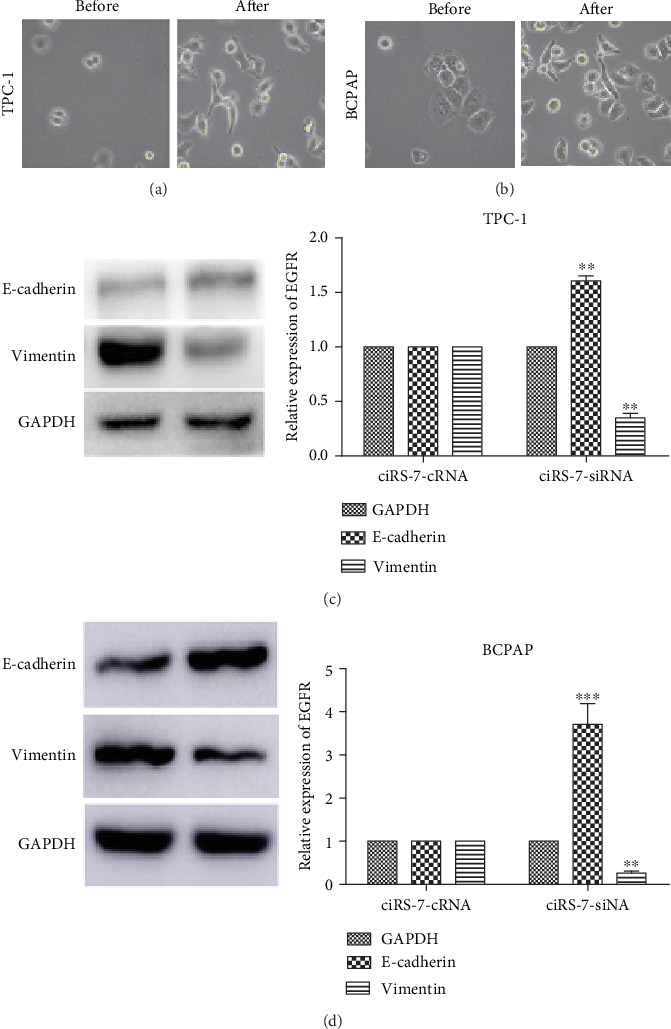
ciRS-7 silencing reduces EMT in TPC-1 cells. Cell lines exhibited a spindle-like, fibroblastic cell morphology after ciRS-7 silencing (a, b). Western blotting assay was conducted to analyze the expression of E-cadherin and vimentin (c, d). ^∗^*P* < 0.05, ^∗∗^*P* < 0.01, and ^∗∗∗^*P* < 0.001.

Since ciRS-7 silencing altered PTC cell morphology such that both cell lines exhibited a spindle-like, fibroblastic cell morphology (Figures 4(a) and 4(b)), we hypothesized that ciRS-7 is involved in the epithelial-mesenchymal transition (EMT) of PTC cells, which is a key event associated with tumor metastasis and invasion. The western blotting results were consistent with the conclusion we expected. Vimentin levels were clearly lower in PTC cells transfected with ciRS-7-siRNA than in negative controls. E-cadherin expression was higher in the ciRS-7-siRNA group than in cells transfected with ciRS-7-cRNA (Figures 4(c) and 4(d)). These results indicate that downregulating ciRS-7 suppresses EMT, which may explain the mechanism of invasion and migration of PTC cells.

### 3.4. ciRS-7 Interaction with miR-7 in PTC

**Figure 5:**
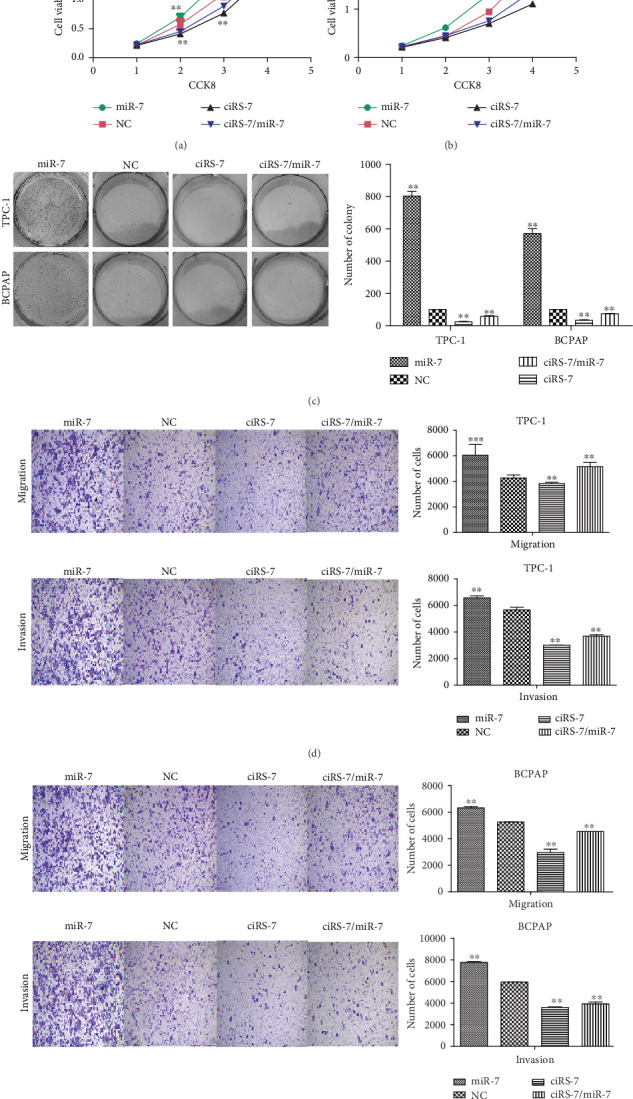
ciRS-7 silencing inhibited the promotion effect mediated by miR-7 knockdown. ciRS-7 silencing neutralized tumor cell proliferation promoted mediated by miR-7 knockdown in TPC-1 and BCPAP (a–c). ciRS-7 silencing neutralized tumor cell proliferation promoted mediated by miR-7 knockdown in TPC-1 and BCPAP (d, e). ^∗^*P* < 0.05, ^∗∗^*P* < 0.01, and ^∗∗∗^*P* < 0.001.

Previous studies have suggested that ciRS-7 could sponge to miR-7. We evaluated the effects of ciRS-7 on regulating miR-7 in PTC cells. Our results confirmed that silencing of ciRS-7 could significantly increase the expression of miR-7 in both TPC-1 and BCPAP (Figure 1(c)). To confirm that whether ciRS-7 inhibits miR-7 for promoting oncogenic potential, CCK-8, colony formation, and transwell assays were performed in the two cell lines with knockdown of miR-7 alone, ciRS-7 alone, or both. CCK-8 and colony formation assay revealed that miR-7 knockdown increased the proliferation ability of TPC-1 and BCPAP, and such effect was partially reversed by silencing ciRS-7 (Figures 5(a)–5(d)). Consistently, ciRS-7 silencing attenuated the increased ability of migration and invasion induced by miR-7 knockdown (Figure 5(e)). Those results indicated that miR-7 is a participant in ciRS-7-induced proliferation, migration, and invasion of PTC cells.

### 3.5. ciRS-7 Regulated EGFR by Sponging of miR-7

**Figure 6:**
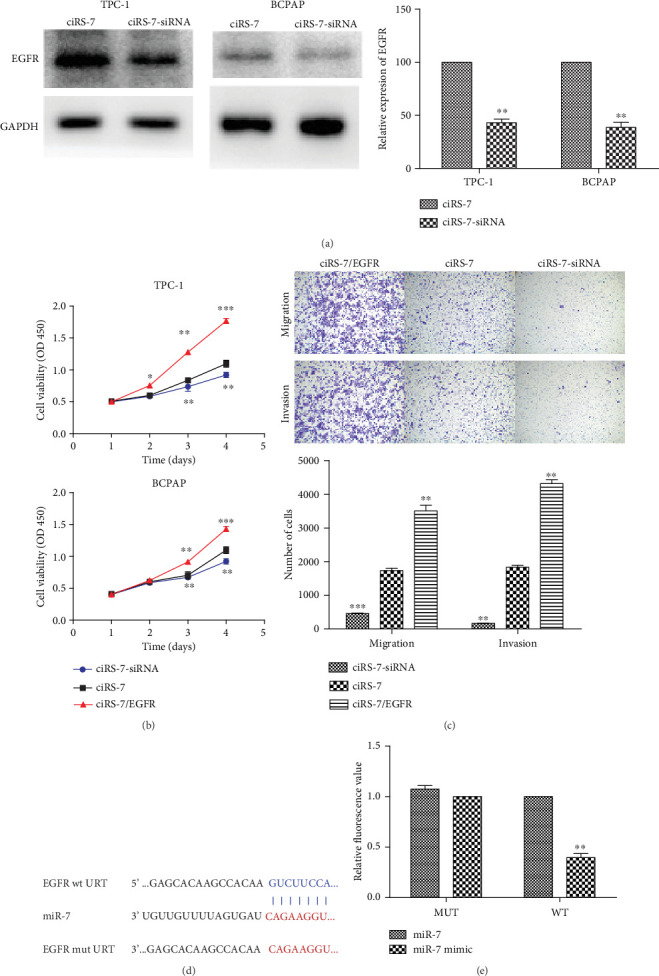
EGFR mediated the promotion effects of ciRS-7/miR-7 axis on PTC cells. The expression of EGFR was decreased by ciRS-7 silencing in TPC-1 and BCPAP (a). Overexpressed EGFR could reverse the effects of ciRS-7 silencing on proliferation, migration, and invasion (b, c). The conserved binding site in 3′UTR of human EGFR mRNA to miR-7 (d). Luciferase system analysis was used to identify the direct binding of miR-7 to 3′UTR of EGFR (e). ^∗^*P* < 0.05, ^∗∗^*P* < 0.01, and ^∗∗∗^*P* < 0.001.

Bioinformatics tools (TargetScan, miRBase, and PicTar) were used to predict the potential target of microRNA-7 (Figure 6(d)). Among the overlap target genes, epidermal growth factor receptor (EGFR) was selected because of its role in cancer development. To further confirm whether miR-7 regulates the expression of EGFR directly, we constructed the wild-type and mutated 3′UTR of human EGFR mRNA and cotransfected with hsa-miR-7 mimic into TPC-1. We found that miR-7 significantly reduced the luciferase intensity in the cells cotransfected with wild-type 3′UTR of EGFR but not the mutant one (Figure 6(e)).

Western blotting assay showed that the expression of EGFR can be decreased by ciRS-7 silencing in TPC-1 and BCPAP (Figure 6(a)). Therefore, we increased the expression of EGFR in PTC cell. The results revealed that overexpressed EGFR could reverse the effects of ciRS-7 silencing on proliferation (Figure 6(b)), migration, and invasion (Figure 6(c)) in vitro. The consistent results suggested that EGFR could be involved in the promotion effects of ciRS-7/miR-7 on PTC cells.

## 4. Discussion

circRNAs have been reported to be participants in the development of various cancers including thyroid cancers. As one of the most extensively investigated circRNA, ciRS-7 is known to modulate proliferation, invasion, and migration in cancers including hepatocellular carcinoma, colorectal, gastric, and lung cancers [14–16]. Experimental data indicated that it could be a valuable biomarker and therapeutic target in esophageal squamous cell carcinoma (ESCC) [17]. In this study, we investigated for the first time the function and mechanism of ciRS-7 in PTC. Our qRT-PCR results showed that ciRS-7 expression was higher in PTC tissues than in normal samples. Overexpressed ciRS-7 contributes to the aggressive clinical-pathological factors such as large tumor size and lymph node metastasis. Moreover, ciRS-7 silencing significantly inhibited PTC proliferation, migration, and invasion in vitro. Overexpressed ciRS-7 promoted PTC proliferation, migration, and invasion. Together with the consistent clinical data, the oncogenic role of ciRS-7 in PTC was significantly supported.

Next, we explored the mechanism by which ciRS-7 affects the development of PTC. After ciRS-7 silencing, PTC cells transformed into spindle epithelium morphologically. We hypothesized that ciRS-7 is involved in the epithelial-mesenchymal transition (EMT) of PTC cells, which was activated during cancer and promoted migration and invasion [18]. We detected the EMT biomarker E-cadherin and vimentin; the level of E-cadherin was higher while vimentin was lower when ciRS-7 was decreased after ciRS-7 silencing. EMT was inhibited by ciRS-7 silencing, which could partly explain how ciRS-7 silencing inhibited migration and invasion in PTC.

Our present study further revealed that ciRS-7 triggered progression of PTC cells via regulating the miR-7/EGFR axis. ciRS-7 contains 74 binding sites for miR-7 and binds densely to it as an inhibitor. Previously, ciRS-7 has been shown to block miR-7 and thereby reactivate genes suppressed by miR-7 in the brain [19], islet cells [20], colorectal cancer [21], lung cancer, and gastric cancer (GC) [22]. miR-7 has been reported to be a tumor suppressor in various tumors. In PTC, miR-7 suppresses cell growth and development via downregulation of many oncogenic signaling pathway such as CKS2/cyclin B2 and cdk1, p21-activated kinase-1 (PAK1). Our data provided another potential downstream target of miR-7 when it is involved in proliferation, migration, and invasion in PTC.

EGFR is a member of the HER family of receptors and is a receptor for members of the EGF family [23]. During tumor progression, specific endogenous ligands activate downstream pathways including the Ras/Raf mitogen-activated protein kinase (MAPK), Jak2/Stat3, and PI3K/AKT pathway [24]. The EGFR/RAF/MAPK pathway is a well-known oncogenic pathway which correlates with metastasis [25]. The JAK/STAT3 signaling could promote invasion and metastasis through activation of key metastasis-promoting genes such as WASF3 [26]. The PI3K/AKT signaling pathway is a crucial player in the regulation of different cellular and molecular processes including cell growth, proliferation, cell motility, and survival in PTC [27]. Furthermore, its therapeutic potential in human cancers has been addressed. In lung cancer, EGFR has become a crucial therapeutic target for patients with non-small-cell lung cancer. EGFR tyrosine kinase inhibitors are the most promising clinical agents as monotherapy for non-small-cell lung cancer [28]. Additionally, data suggest that blocking EGFR can effectively increase the antitumor activity of selumetinib in triple-negative breast cancer, which may be related to the effect of this combination on the activation of extracellular signal-regulated kinase 1/2 and AKT [29]. In PTC, EGFR was demonstrated to upregulate in about 55% of PTCs and is correlated with aggressive behaviors of PTC [30].

Using publicly available algorithms (TargetScan, miRanda, and PicTar), we identified EGFR as a potential target of miR-7. Luciferase reporter assay further demonstrated their interaction. Moreover, we found that the protein level of EGFR is decreased distinctly in ciRS-7-silencing cells. EGFR overexpression significantly reversed the effects of ciRS-7 silencing on PTC cell proliferation, migration, and invasion, indicating that EGFR was involved in the progression induced by the ciRS-7/miR-7 axis in PTC. We believed that ciR-7 inhibited proliferation, migration, and invasion of PTC by targeting the miR-7/EGFR axis.

## 5. Conclusion

Our results indicated that ciRS-7 promotes the genesis and development of PTC by increasing the proliferation and migration of PTC cells. Regulation of the ciRS-7/miR-7/EGFR axis is a crucial molecular mechanism in PTC, and this pathway may be a novel target for the diagnosis and therapy of this cancer.

## Figures and Tables

**Table 1:** Correlation of clinicopathological features and ciRS-7 expression in PTC tissues.

Characteristics	No. of cases	*P* value
Gender		
Female	14	0.15
Male	3	
Age		
<50	12	0.20
>50	5	
Cancer size		
<1 cm	7	0.022
>1 cm	10	
Lymph node metastasis		
Yes	5	0.015
No	12	

## Data Availability

The raw data required to reproduce these findings cannot be shared at this time as the data also forms part of an ongoing study.

## Supplementary Materials

Supplementary Materials To explore the functions of ciRS-7, two short interfering (ciRS-7-s1 and ciRS-7-s2) vectors were transfected into TPC-1 cell line. The effects of ciRS-7 silencing on PTC is shown.
