# Supplementary material for: ciRS-7 Promotes the Proliferation and Migration of Papillary Thyroid Cancer by Negatively Regulating the miR-7/Epidermal Growth Factor Receptor Axis
Source: Biomed Res Int. 2020 Jun 21;2020:9875636. doi: 10.1155/2020/9875636 (PMC7327576; doi:10.1155/2020/9875636)
Supplement: Supplementary Materials — To explore the functions of ciRS-7, two short interfering (ciRS-7-s1 and ciRS-7-s2) vectors were transfected into TPC-1 cell line. The effects of ciRS-7 silencing on PTC is shown. [file 9875636.f1.pdf]

Supplementary Materials for

CiRS-7 reduces the proliferation and migration of papillary thyroid cancer by negatively regulating the miR-7/epidermal growth factor receptor axis

Jun-ya Han[1,2,3#], Si Guo[4,5#], Na Wei[1,2], Xiangyu Tian[1,2], Chao Chen[1,2], Sen Qiu[1,2], Tong Wang[1,2], Qiankun Xiao[1,2], Jingjing Xu[*1,2,3], Kui-sheng Chen[*1,2,3]

1 Department of Pathology, The First Affiliated Hospital of Zhengzhou University, No. 1 Jian She Dong Avenue, Zhengzhou, People's Republic of China

2 Henan Key Laboratory of Tumor Pathology, No. 1 Jian She Dong Avenue, Zhengzhou, People's Republic of China

3 Academy of medical sciences, Zhengzhou University, No. 100 Ke Xue Avenue, Zhengzhou, People's Republic of China

4 Department of Medical laboratory , Henan Provincial People's Hospital, Zhengzhou, Henan, China

5 Department of Medical laboratory of Central China Fuwai Hospital, Central China Fuwai Hospital of Zhengzhou University, Zhengzhou, Henan, China

Short Title: Affections of ciRS-7 in papillary thyroid cancer

#These authors contributed equally to this work

*Correspondence to: Jingjing Xu. The First Affiliated Hospital of Zhengzhou University, No. 1 Jian She Dong Avenue, Zhengzhou, 450002, China. E-mail: happyxujingjing@163.com;

Kuisheng Chen. The First Affiliated Hospital of Zhengzhou University, No. 1 Jian She Dong Avenue, Zhengzhou 450002, China. E-mail: chenksh2002@163.com

The PDF file includes:

Materials and Methods

Fig 1 The effects of ciRS-7 silencing on thyroid carcinoma cell proliferation by CCK8 assay and colony formation assay.

Fig 2 The effects of ciRS-7 silencing on thyroid carcinoma cell migration by wound

healing assay.

Fig3 The effects of ciRS-7 silencing on thyroid carcinoma cell migration and invasion by transwell assay.

Materials and Methods

Cell transfection

The human thyroid cancer cell line TPC-1 was acquired from American Type Culture Collection (Manassas, VA, USA). Small interfering RNAs against ciRS-7 (ciRS-7-s1 and ciRS-7-s2) and negative control siRNA were synthesized by Genema (Shanghai, China). Then TPC-1 was transfected with the ciRS-7 silencing vector at 50 nM by using Lipofectamine 2000 (Invitrogen).

Cell proliferation assay

Cell proliferation was determined using a CCK-8 assay kit (Dojindo, Kumamoto, Japan) according to the manufacturer's instructions. Cells were seeded (100 μL; 2×10^3 cells per well) into 96-well plates and CCK-8 was added at 0, 24, 48, 72h. Absorbance was measured at 450 nM using an enzyme-labeling instrument (Thermo Fisher Scientific) afterward. For colony formation assay, cells were seeded (500 cells per well) into six-well plates and incubated for 10 days. The colonies were stained and observed under a microscope.

Scratch test

Cells were seeded into six-well plates to detect the wound healing capabilities. When the cells covered 80–90% of the dish, a 100 μL pipette tip was used to make four scratches at the same width in each well. PBS was used to wash away the cells removed during scratching. Next, the cells were cultured in fresh culture medium in an incubator. An inverted microscope was used to observe the migration distance of cells into the scratch area at 0, 6, 24, and 36 h. The assay was repeated three times.

Transwell migration and invasion

Transwell migration assays were performed using a Transwell chamber (Corning, China). Transfected cells in serum-free medium were seeded in the upper transwell chamber and 500ųl RPMI solution that included 20% fetal bovine (FBS) serum were added into the lower transwell chamber. After being cultured for 24 hours, cells were fixed with paraformaldehyde and stained using crystal violet. An inverted light microscope was used to quantify the migrative cells and views were randomly observed to obtain the average results. Invasion assay was conducted in accordance with the above procedures except that the bottom membranes were coated with the diluted Matrigel.

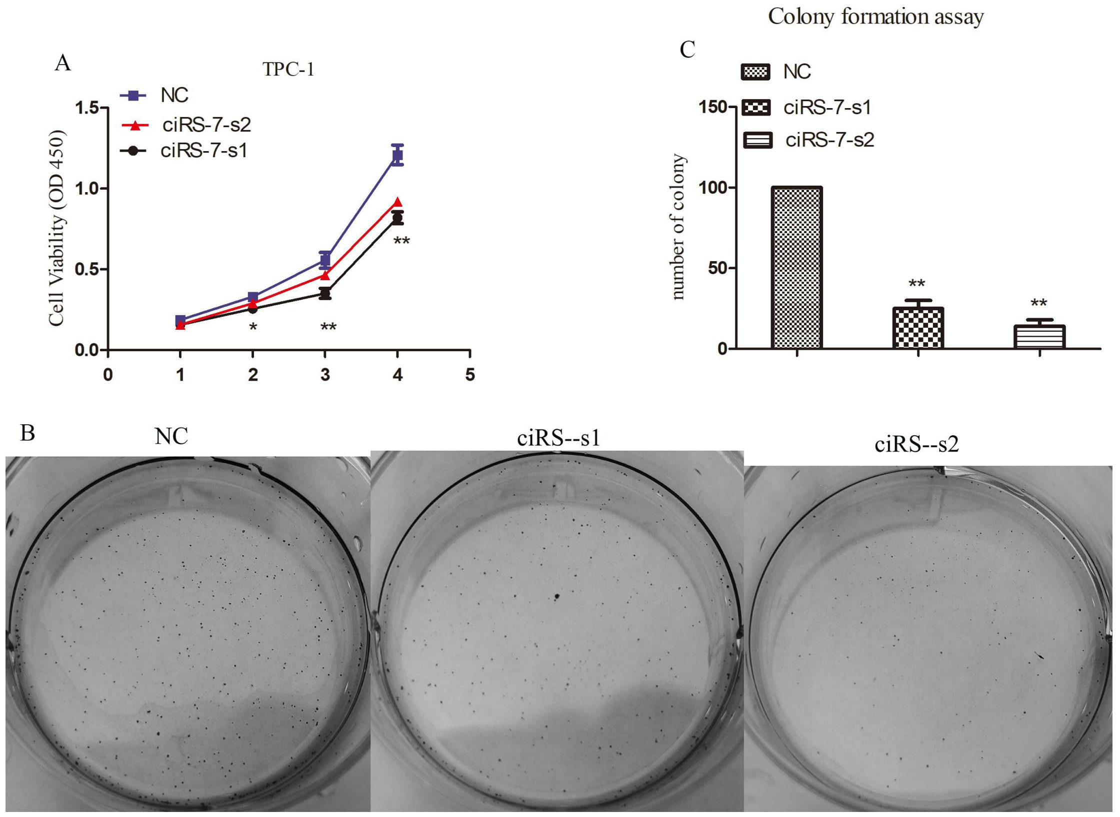

Fig 1 The effects of ciRS-7 silencing on thyroid carcinoma cell proliferation by CCK8 assay and colony formation assay.

Cell proliferation of thyroid carcinoma cells TPC-1 after ciRS-7 siRNAs or negative control siRNA transfection was evaluated by CCK8 assay (A). Representative images of colony formation (B) assay using TPC-1 and quantification analysis of colony numbers (C). *$p < 0.05$, **$p < 0.01$, ***$p < 0.001$.

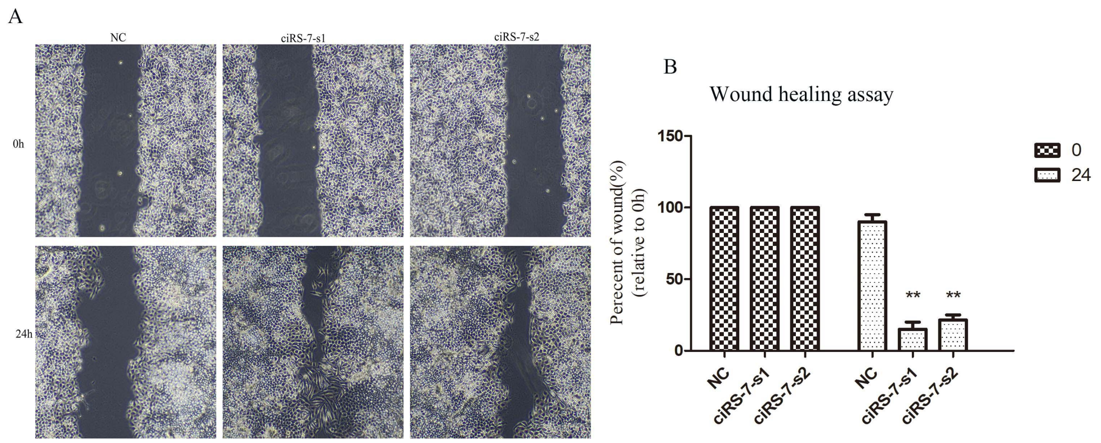

Fig 2 The effects of ciRS-7 silencing on thyroid carcinoma cell migration by wound healing assay.

Cell migration of TPC-1 cell after two ciRS-7-siRNA (ciRS-7-s1 and ciRS-7-s2) or negative control siRNAs transfection was evaluated by wound healing assay. *p < 0.05, **p < 0.01, ***p < 0.001.

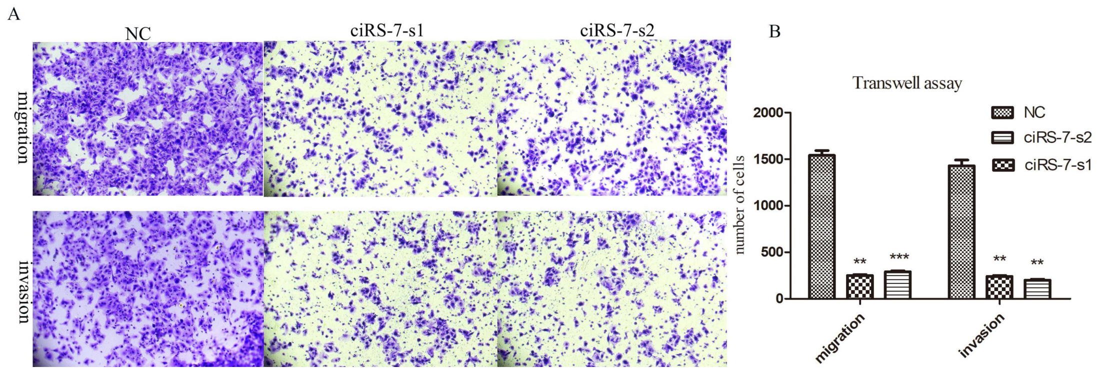

Fig3 The effects of ciRS-7 silencing on thyroid carcinoma cell migration and invasion by transwell aassay.

Transwell invasion assays were conducted to assess the cell migration and invasive abilities of thyroid carcinoma cells TPC-1 after ciRS-7 siRNAs or negative control siRNAs transfection. *p < 0.05, **p < 0.01, ***p < 0.001.
